# When Does Support to Adult Children Negatively Affect the Subjective Well-Being of Older Japanese?

**DOI:** 10.1093/geroni/igab046.1626

**Published:** 2021-12-17

**Authors:** Erika Kobayashi

**Affiliations:** Tokyo Metropolitan Institute of Gerontology, Itabashi-ku, Tokyo, Japan

## Abstract

Providing time and money to adult children may enhance perceived usefulness and consequently the subjective well-being (SWB) of older parents. However, non-reciprocal relationships with children and conflicts with leisure activities could negatively affect parents’ SWB. It was hypothesized that a substantial amount of support to children would be associated with lower SWB when older parents (a) had a low expectation of receiving long-term care from the children, and (b) were engaged in hobbies/learning activities. Life satisfaction and depressive symptoms measured as SWB were predicted based on the Generalized Estimating Equations, using panel data (2012-2017) with a nationwide representative sample of Japanese adults aged 60 years and older (1,212 parents). Providing child-rearing support (i.e., grandchild care) of 30 hours or more per month was positively associated with SWB regardless of conditions (a) and (b). Hypothesis (b) was partially supported: providing financial support enhanced depressive symptoms among older adults with hobbies/learning.

